# Virtual Planning of a Complex Three-Part Bimaxillary Osteotomy

**DOI:** 10.1155/2017/8013874

**Published:** 2017-11-28

**Authors:** Chiara Di Blasio, Marilena Laura Anghinoni, Alberto Di Blasio

**Affiliations:** University of Parma, Parma, Italy

## Abstract

In maxillofacial surgery, every patient presents special problems requiring careful evaluation. Conventional methods to study the deformities are still reliable, but the advent of tridimensional (3D) imaging, especially computed tomography (CT) scan and laser scanning of casts, created the opportunity to better understanding the skeletal support and the soft tissue structures. Nowadays, virtual technologies are increasingly employed in maxillofacial surgery and demonstrated precision and reliability. However, in complex surgical procedures, these new technologies are still controversial. Especially in the less frequent cases of three-part maxillary surgery, the experience is limited, and scientific literature cannot give a clear support. This paper presents the case of a young patient affected by a complex long face dentofacial deformity treated by a bimaxillary surgery with three-part segmentation of the maxilla. The operator performed the surgical study completely with a virtual workflow. Pre- and postoperative CT scan and optical scanning of plaster models were collected and compared. Every postoperatory maxillary piece was superimposed with the presurgical one, and the differences were examined in a color-coded map. Only mild differences were found near the osteotomy lines, when the bony surface and the teeth demonstrated an excellent coincidence.

## 1. Background

A satisfying outcome in orthognathic surgery depends on the surgical technique and the accuracy of the orthodontic-surgical treatment plan. Surgical decisions are based on complex clinical and instrumental data. Until today, at the end of the presurgical orthodontic treatment, the surgeon simulates the desired bony movements on the casts and creates dental splints to be employed during the surgery. This process demonstrated reliability both for common clinical situations [1] and for the most difficult deformities [2, 3]; however, 3D imaging new technologies ensure a number of advantages for the clinician. It is nowadays possible to create a precise “virtual patient” combining data from three-dimensional scan of the maxillofacial skeleton [4–10], 3D scan of the dental casts or intraoral scanning, and, when necessary, stereophotogrammetry [11]. Several scientific papers have described these new procedures for the surgical planning [12–21]; these modern technologies do not add any new data but make data easier to interpret allowing a deeper understanding of the problems and a very accurate simulation of the surgery. In 2010, Tucker [22] demonstrated that the virtual surgery accurately reproduces surgical movements in all the planes of the space for both one- and two-jaw surgeries. For these reasons, this modern approach is rapidly emerging and increasingly important in maxillofacial surgery. This approach, compared to conventional planning, ensures several advantages: (1) The accuracy in identifying some fine details (i.e., maxillary cant or asymmetry in mandibular angles) that may be undetected by the simple clinical examination [12, 13, 23]. (2) Aesthetical appearance, important in children [24] and indeed the main goal in maxillofacial surgery of adults. The freedom for the surgeon to easily simulate and compare different solutions choosing the best one for the patient is precious. (3) Maintaining the condylar health and function is mandatory in maxillofacial surgery [25, 26] although sometimes it can be difficult in the complex situations [27, 28]. The new technologies ensure high degree accuracy in transferring virtual surgical plan on the patient avoiding condylar displacements [29–31]. (4) Virtual planning provides new possibilities in surgical precise positioning of the bones when associated with navigation systems [4, 15, 32]. In the near future, other useful tools will be soon available such as 3D cephalometry. Even if most authors still prefer the traditional 2D cephalometric analysis [33–35], also for radioprotection considerations [36], 3D cephalometry is getting more and more accurate, and in a near future, very promising [37]. Moreover, the increasing accuracy of the software in predicting soft tissue changes will allow a more accurate planning of the aesthetic outcome [38, 39]. The surgical simulation, made upon the “virtual patient” data, are transferred to the real patient using surgical splints, fabricated by the computer-aided manufacturing (CAD/CAM) techniques [40–42]. Several software are dedicated to the 3D virtual planning [32]; the most frequently employed are SimPlant O&O (Materialise, Leuven, Belgium) and Dolphin 3D (Dolphin Imaging and Management Solutions, Chatsworth, CA, USA). As previously stated, it is possible to say that the virtual surgery accurately reproduces surgical movements in all the planes of the space for both one- and two-jaw surgeries [22]; however, this might not be the case for even more complex surgical procedures. These complex procedures are more difficult and less frequent, and in these cases, the scientific literature is less helpful. Especially in three-part maxillary surgery, it may be difficult to reassemble the maxillary fragments in 3D, and the predictability of the system may be more uncertain [13–19]. In this case report, the authors present a complex dentofacial deformity. The special complexity was due to the craniofacial long face disharmony requiring a bimaxillary surgery with a three-part segmentation of the maxilla. After a conventional planning of the orthodontic treatment, the patient was completely managed by the new virtual technologies for the planning of the surgery.

## 2. Case Presentation

**Figure 1:**
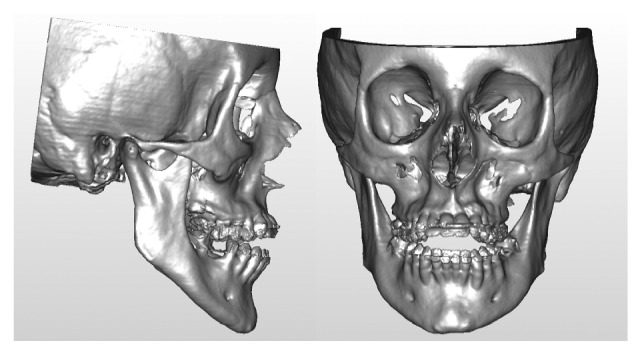


**Figure 2:**
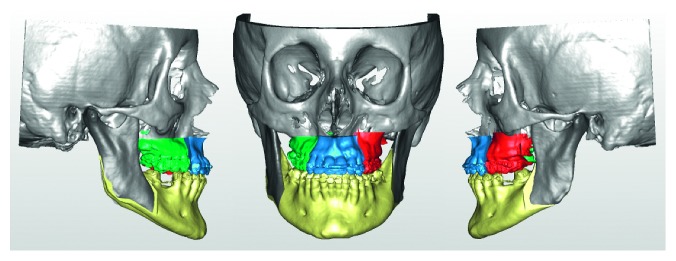


**Figure 3:**
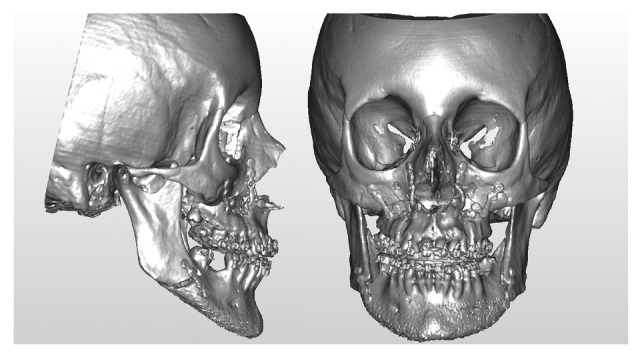


A 19-year-old female was referred to the Maxillofacial Department of Parma University Hospital for a complex long face dentofacial deformity. The face of the patient demonstrated an important vertical height excess with lips incompetence and a severe dental anterior open-bite malocclusion. As well known, correcting the vertical dimension of the face always involves difficult decisions, for both long and short face situations [43–46]. The orthodontic presurgical treatment was decided by the usual approach studying the face, the occlusion, the casts, and the 2D cephalometry. The orthodontist was required to align and derotate the teeth avoiding any correction of the open bite, creating an ideal continuous lower arch and a segmented upper one, allowing for a three-piece maxillary surgery. At the end of the orthodontic treatment, the patient underwent a presurgical final evaluation by the new virtual surgery technologies. The following instrumental exams were collected: CT scan of the craniomaxillofacial complex and laser scan of the new casts. For the CT scan, a multislice CT Emotion 6 (Siemens Co., München, Germany), and for the casts, a S600 ARTI scanner (Zirkonzahn S.r.L., Gais, Italy) was employed. The dicom data from the CT scan were evaluated by the software SimPlant O&O, reconstructing the skull and matching the low-resolution teeth image from the CT scan with the high-resolution laser scan of the casts (Figure 1). The software was then employed for the simulation of the maxillary three-part and the bilateral sagittal split osteotomy (BSSO) of the mandible (Figure 2). The particular challenge of the case was in the maxillary surgery: it is quite difficult to simulate a three-piece maxillary surgery on both the casts and the computer. In this long face patient, the lateral pieces of the maxilla underwent an upper repositioning while the anterior portion was moved anteriorly with a minimal vertical correction. The total advancement of the maxillary complex was 4 mm. In the BSSO simulation, the mandible was advanced about 2 mm and rotated upward, allowing the facial height correction (Figure 2). After the virtual planning was decided and the relative simulation was performed, the surgical splints were fabricated by the CAD/CAM technology, and the patient underwent surgery. Three months after the surgery, a complete set of new 3D data was collected and evaluated by the same 3D software (Figure 3). The coincidence between the virtual program and the final surgical result for the anterior and lateral pieces of the maxilla by means of 3D superimpositions was evaluated. The same software SimPlant O&O was allowed to superimpose and compare the 3D objects. The superimposition was made comparing the surgical planning (Figure 2) to the final outcome of the treatment (Figure 3). The superimposition was regulated to show in color-coded maps the differences between the two 3D objects: from green color meaning perfect coincidence to violet color meaning a difference of 3 millimeters.

**Figure 4:**
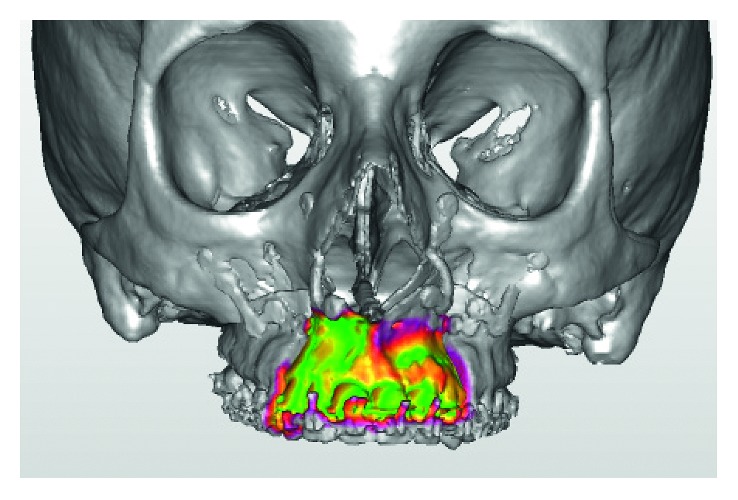


The anterior portion of the color-coded map demonstrated a good correspondence between the prediction and the final result (Figure 4). A small red-violet area at the upper limit of the left border was present showing, only in this point, a 2-3 mm difference. Since no difference exists on the bone surface and the teeth, it was possible to assume that the difference is limited to the osteotomy lines.

**Figure 5:**
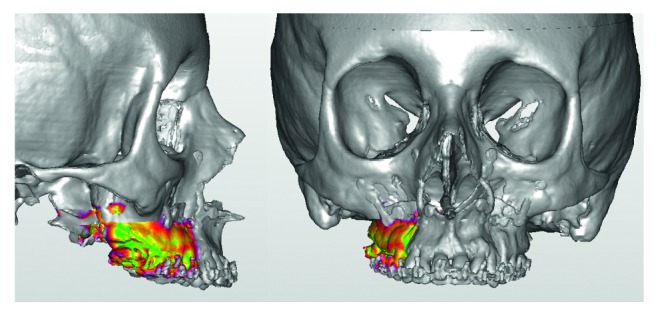


In the right portion, also for the right maxillary piece, the large green area demonstrated an excellent coincidence (Figure 5). Only minor discrepancies are visible on the distal edge of the molar and on the osteotomy lines.

**Figure 6:**
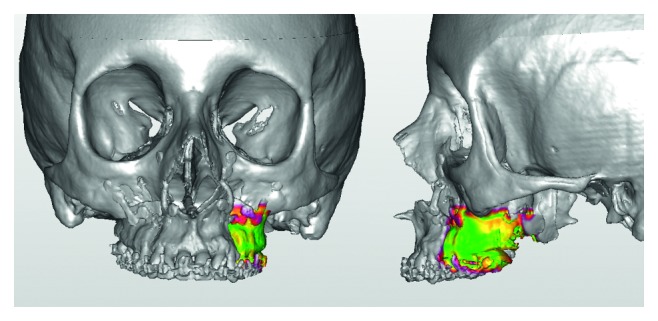


In the left portion, the outcome was similar (Figure 6) showing good correspondence between the 3D models except for the osteotomy lines.

## 3. Discussion and Conclusions

Virtual planning for maxillofacial surgery is increasingly emerging as a useful procedure in the clinical routine. In our experience, it represents now a routine tool for both single- and double-jaw surgeries. However, virtual surgery planning is less common in cases of maxillary segmentation, due to the lower frequency of the pathologies requiring this procedure and the difficulties in programming the surgery on the computer. A two-piece maxillary surgery is generally limited to special clinical situations, that is, in the transversal defect [47] when a three-piece maxillary surgery is indicated in increased vertical dimension deformities. This case report was particularly complex because it required a three-part segmentation. The three-part maxillary surgery with superior repositioning allows a mandibular counterclockwise rotation correcting then the facial vertical excess. The amount of the superior repositioning and the rotation has to be carefully planned. Virtual technologies ensured to the surgeon the considerable advantage to compare, easily and in short time, different hypotheses coming back to the original bone position by a simple click on the mouse. This possibility obviously also exists in the conventional method, but handling several times a three-piece maxillary cast may add further bias to the procedure. The almost perfect coincidence between presurgical and final 3D imaging demonstrated that the surgical planning made on the computer might be correctly transferred on the patient. In the authors' opinion, these procedures should become the best choice even in segmented surgery.

## Abbreviations

CT: Computed tomography
3D: Tridimensional.
